# Understanding genetics, sex and signaling: Implications of sex-dependent APOE4-neutrophil-microglia interactions for Alzheimer’s and tauopathies

**DOI:** 10.1038/s41392-024-01967-1

**Published:** 2024-09-23

**Authors:** Natja Haag, Juliane Bremer, Hans Zempel

**Affiliations:** 1 Institute for Human Genetics and Genomic Medicine, University Hospital RWTH Aachen, Aachen, Germany; 2 Institute of Neuropathology, University Hospital RWTH Aachen, Aachen, Germany; 3 Institute of Human Genetics, Faculty of Medicine and University Hospital Cologne, University of Cologne, Cologne, Germany; 4 Center for Molecular Medicine Cologne (CMMC), University of Cologne, Cologne, Germany; 5 Department of Nuclear Medicine, Faculty of Medicine and University Hospital Cologne, University of Cologne, Cologne, Germany

**Keywords:** Diseases of the nervous system, Molecular medicine

A recent study published in *Nature Medicine* uncovers a novel sex-dependent mechanism involving the apolipoprotein E ε4 (APOE4) and in particular neutrophils and their signaling to microglia, which then would mediate cognitive decline in late-onset Alzheimer’s disease (AD).^1^ We here put the main findings into perspective with current knowledge about *APOE4* genotype and neutrophils in the pathogenesis of AD and related tauopathies, and discuss future research directions and therapeutic implications.

*APOE4* homozygosity is the strongest genetic risk factor for AD, especially in females. While *APOE4* homozygotes are at very high risk for developing AD, appear to be less sensitive to the beneficial effects of estrogen and show altered sex-hormone responses, this study now sheds light on an immune cell-mediated mechanism that conveys increased risk also to female heterozygotes.^1^

Neutrophils (or neutrophilic granulocytes) are most abundant in bacterial or fungal infections or acute tissue necrosis. As part of the innate immune system, they are crucial in the defense against microorganisms. More recently, neutrophils were shown to play an important role in neurodegenerative diseases, especially Alzheimer’s disease (AD), which is, however, histopathologically not characterized by overt granulocytic infiltrates. Elevated neutrophil-lymphocyte ratios are observed in the blood of AD patients and interpreted as a sign of systemic inflammation. More recent findings suggest a local role within the brain. In AD mouse models, neutrophil adhesion to the wall of brain capillaries has been shown to reduce cortical blood flow and impair memory function. Notably, these effects were reversible upon administration of antibodies against the neutrophil marker Ly6G.^2^ In addition to intravascular adhesion of neutrophils in cortical capillaries, they also extravasate to infiltrate the brain parenchyma to colocalize with amyloid-beta plaques, especially during the onset of memory loss,^3^ suggesting a crucial role for neutrophils in AD.

Like neutrophils, microglial cells are cellular components of the innate immune system but are specific to the central nervous system. Microglial cells are thought to play a crucial role in the neuropathology of AD, where they exhibit both, a pro-inflammatory - in part detrimental, in part beneficial - as well as a beneficial amyloid-beta clearing function. Recent studies illustrate how microglial activation differs significantly depending on sex, hormone levels, and genetic background. Female microglia actually exhibit more pronounced pro-inflammatory profiles than those of males under neurodegenerative conditions.

**Fig. 1:**
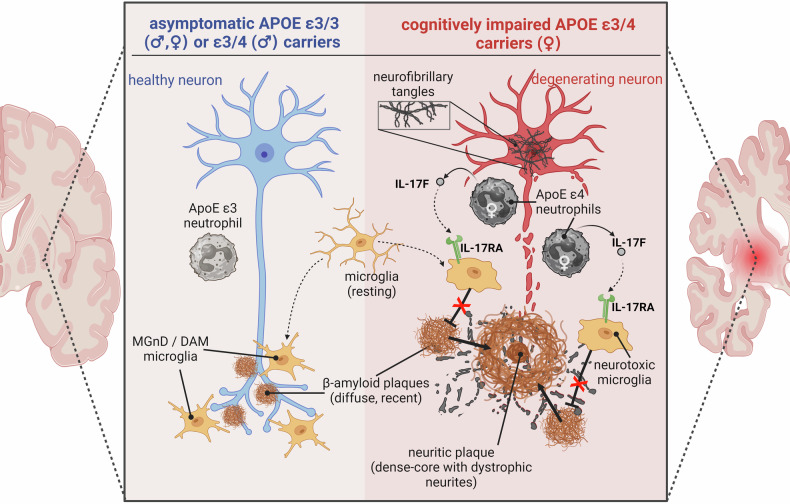
Sex-dependent neutrophil-mediated activation of microglia via IL-17F to IL-17RA signaling results in impaired amyloid-beta plaque clearance and enhanced neurodegeneration in female *APOE ε3/4* carriers. Left: Physiological activation of microglia towards a neuroprotective and plaque-clearing state (MGnD/DAM microglia) in asymptomatic aged individuals (e.g. *APOE ε3/3* genotype or male *APOE ε3/4* carriers). Right: Pathological signaling in female *APOE ε3/4* carriers of neutrophils to microglia resulting in microglia unable to clear amyloid-beta plaques, which in turn contributes to the formation of dense-core and neuritic plaques, and neurodegeneration. Created with BioRender.com

In the new study discussed here, Rosenzweig et al. established a novel direct interaction between these two cellular components of the innate immune system, neutrophils, and microglial cells within the brain parenchyma in patients with AD. The authors demonstrate that APOE controls a sex-dependent immunosuppressive phenotype of IL-17 expressing neutrophils that upregulated the immunosuppressive cytokines IL-10 and TGF-beta and immune checkpoints, including LAG3 and PD-1. They further dissect this interaction by showing that IL-17F is released from neutrophils and binds to IL-17RA on microglial cells. This IL-17 signaling suppresses the induction of a neurodegenerative microglial phenotype - so-called neurodegenerative microglia (MGnD), also known as disease-associated microglia (DAM), which in turn has been shown to combat neuronal dysfunction and cognitive impairment (Fig. 1). This mechanism acts in both sexes with the impact being greater in *APOE* ε3/4 female carriers. APOE4 depletion from neutrophils actually reduced this immunosuppressive phenotype of neutrophils and restored the microglial response to neurodegeneration, limiting plaque pathology. Hence, this study also provides important insights into the mechanism of why the *APOE4* allele is such a strong genetic risk factor for AD, especially in females. Blocking the IL-17-mediated interplay between neutrophils and microglia improved cognitive function in AD mouse models. While in general AD is most commonly multifactorial and difficult to tackle therapeutically, these findings suggest that especially female *APOE4* carriers with AD could benefit from targeting these neutrophil-microglia interactions, specifically the IL-17F/IL-17RA axis, possibly in combinatorial therapeutic approaches to reduce cognitive decline. Nonetheless, this study underscores the critical need for sex- and (*APOE*) genotype-specific approaches in AD therapy and is a step towards tailored personalized therapeutic approaches in AD/neurodegeneration, and the identified neutrophil subset and associated molecular signatures could serve as biomarkers for early detection or monitoring of AD progression in at-risk populations.

Now, validating these findings in larger human cohorts across different ethnic populations is crucial for clinical translation, in particular when thinking of tailored medicine. Investigating the long-term consequences of modulating neutrophil-microglia interactions on overall brain health and function is necessary. Exploring potential synergies between targeting these interactions and existing AD therapies, such as anti-amyloid-beta approaches, could yield more effective treatment strategies. Determining whether early intervention targeting these pathways could prevent or delay AD onset in at-risk individuals, particularly female *APOE4* carriers, must be addressed.

Rosenzweig et al. demonstrate that neutrophils and microglia physically interact in the brain parenchyma of AD patients.^1^ However, given the observed role of intravascular neutrophils in brain capillaries^2^ and the proposed systemic effects of elevated systemic inflammation with increased neutrophil to lymphocyte ratio, signaling across the blood-brain barrier or systemic/humoral effects are alternative possibilities. In fact, IL-17 has been suggested as an important mediator of the gut-brain axis, and IL-17 has also been shown to impact neurons directly (as previously reviewed^4^). Therefore, the anti-IL-17F treatment might restore cognitive function in AD disease models not only through the neutrophil-microglia axis but also in part by affecting the role of IL-17 on neurons.

Importantly, these APOE- and IL-17-driven neutrophil-microglial interactions might extend to other neurodegenerative diseases, including tauopathies characterized by aggregation of phosphorylated TAU protein without amyloid-beta pathology. Tauopathies include frontotemporal dementia (FTD), progressive supranuclear palsy (PSP), and corticobasal degeneration (CBD). Like AD, they also feature prominent neuroinflammatory processes. Genetic causes are diverse, with more genes causative for tauopathy to be identified.^5^ Tauopathies show sex-based differences in prevalence or severity: Across various tauopathies, women generally exhibit higher regional TAU pathology compared to men. The sex-dependent impact of the APOE genotype in neutrophil-induced microglial transition to MGnD now identified may also influence the disease course of tauopathies. Hence, targeting the IL-17-associated neutrophil-microglia interplay might be beneficial across a wider spectrum of neurodegenerative disorders.

In conclusion, the research by Rosenzweig et al., viewed through the lens of broader literature connecting neuroinflammation, sex differences, and *APOE4* implications, offers a framework for understanding cognitive impairment in AD and other neurodegenerative disorders such as tauopathies. These findings prompt a re-evaluation of existing therapeutic strategies and highlight the necessity for sex- and genotype-based considerations in future research endeavors across the spectrum of neurodegenerative diseases. By recognizing the shared pathological mechanisms and the influence of sex-dependent factors, we can work towards more targeted and effective treatments for these devastating disorders.
